# Validation of Player and Ball Tracking with a Local Positioning System

**DOI:** 10.3390/s21041465

**Published:** 2021-02-20

**Authors:** Patrick Blauberger, Robert Marzilger, Martin Lames

**Affiliations:** 1 Chair of Performance Analysis and Sports Informatics, Technical University Munich, 80992 Munich, Germany; martin.lames@tum.de; 2 Fraunhofer Institute for Integrated Circuits IIS, 90411 Nuremberg, Germany; robert.marzilger@iis.fraunhofer.de

**Keywords:** validity, accuracy, local positioning system, player tracking, ball tracking, position, speed, acceleration, team sports

## Abstract

The aim of this study was the validation of player and ball position measurements of Kinexon’s local positioning system (LPS) in handball and football. Eight athletes conducted a sport-specific course (SSC) and small sided football games (SSG), simultaneously tracked by the LPS and an infrared camera-based motion capture system as reference system. Furthermore, football shots and handball throws were performed to evaluate ball tracking. The position root mean square error (RMSE) for player tracking was 9 cm for SSCs, the instantaneous peak speed showed a percentage deviation from the reference system of 0.7–1.7% for different exercises. The RMSE for SSGs was 8 cm. Covered distance was overestimated by 0.6% in SSCs and 1.0% in SSGs. The 2D RMSE of ball tracking was 15 cm in SSGs, 3D position errors of shot and throw impact locations were 17 cm and 21 cm. The methodology for the validation of a system’s accuracy in sports tracking requires extensive attention, especially in settings covering both, player and ball measurements. Most tracking errors for player tracking were smaller or in line with errors found for comparable systems in the literature. Ball tracking showed a larger error than player tracking. Here, the influence of the positioning of the sensor must be further reviewed. In total, the accuracy of Kinexon’s LPS has proven to represent the current state of the art for player and ball position detection in team sports.

## 1. Introduction

The analysis of sports performance in training and competition often relies on automatic position detection. Various decisions are based on metrics derived from player tracking variables of these systems. Positional data are used for monitoring players’ training loads [1,2], activity profiles [3] or tactical performance analysis [4,5]. Additionally, positional information about the ball can be used for further analysis, such as the integration of ball possession [6,7].

To acquire positional information, three methods are commonly used in sports practice, as well as scientific investigations: Global positioning systems (GPS), local positioning systems (LPS) and semi-automatic video tracking systems (VID) [1,8,9]. Regardless of the tracking method, an individual, sport-specific validation of each system is necessary to allow a proper interpretation of the position information [8,10,11,12,13]. Much effort can already be found in the validation of GPS [8,14,15], LPS [8,9,10,11,15,16,17,18,19,19] and VID [8,20]. Different sports like football [8,15,20,21,22,23], handball [24], ice hockey [18] or general sport-specific settings [8,9,17,20] are evaluated. Furthermore, some studies investigated comparability of results obtained from the three methods [8,25,26]. For some of these systems, the continuous positional tracking of balls is possible. However, none of the aforementioned validation studies accounted for the accuracy of ball tracking. The tracking of balls is solely validated in separate studies. Seidl et al. investigated tracking accuracy of a RedFir radio-based tracking system and compared the results to lighting gates. A mean bias of 2.6% was found, meaning a slight overestimation of the ball speed measured by the LPS [27]. The continuous accuracy of ball tracking was not assessed, highlighting a shortcoming of lightning gates. Witt and colleagues investigated the detection of single ball contacts with help of a LPS. Ball tracking turned out to be sufficient for detection of events [28].

Generally, the methodology of validation studies of LPS in sports turned out to be complex and therefore requires many specific considerations. An individual, sport-specific evaluation of each system is necessary [11,12,13]. For validation purposes, the usage of a proper gold standard reference system is necessary to validate instantaneous position, speed and acceleration [12]. Further critical points are the adaptation of filter parameters or the correction for gait patterns [8,9,20].

Kinexon’s LPS is a widespread and commercially available system in the segment of sports tracking. This system is used in the first division of the German handball national league and also the Velux EHF Final4 to record matches since the 2019/2020 season [7]. A recent study from Fleureau et al. looked at the validity of peak speed and acceleration of Kinexon’s LPS in handball specific movements [24]. They compared values to the results of simultaneous motion capture and concluded an acceptable validity. Care should be taken near the border of the playing field [24]. Alt et al. investigated the running based validity of Kinexon’s LPS in a sport-specific circuit. They found a good to moderate tracking validity with better results in outdoor tracking [9]. Hoppe et al. focused on validity and reliability of a GPS and Kinexon’s LPS by comparing the results of both systems to timing gate reference values. They found superior overall LPS values, although more outlier measurement errors occurred [10]. A validation study including player or ball position measurements with Kinexon’s LPS and an infrared camera-based criterion reference system was not found in the literature.

Therefore, the aim of this study was the validation of Kinexon’s player and ball tracking capabilities, specifically for applications in handball and football. To achieve this validation, the LPS system’s position tracking was compared to an infrared camera-based reference system with superior accuracy.

## 2. Materials and Methods

### 2.1. Participants

Eight adolescent male players from a professional handball club (age: 14.9 ± 1.2 years, height: 1.8 ± 0.1 m, weight: 75.5 ± 5.0 kg) participated in this study. Prior to the study, all players received verbal and written information about purpose, procedure and requirements of the test. All captured data was anonymized. The protocol accorded to the ethic standards of the Technical University of Munich and was in accordance with the Declaration of Helsinki. Each participant and their parents gave written informed consent to participate in this study.

### 2.2. Tested System

**Figure 1:**
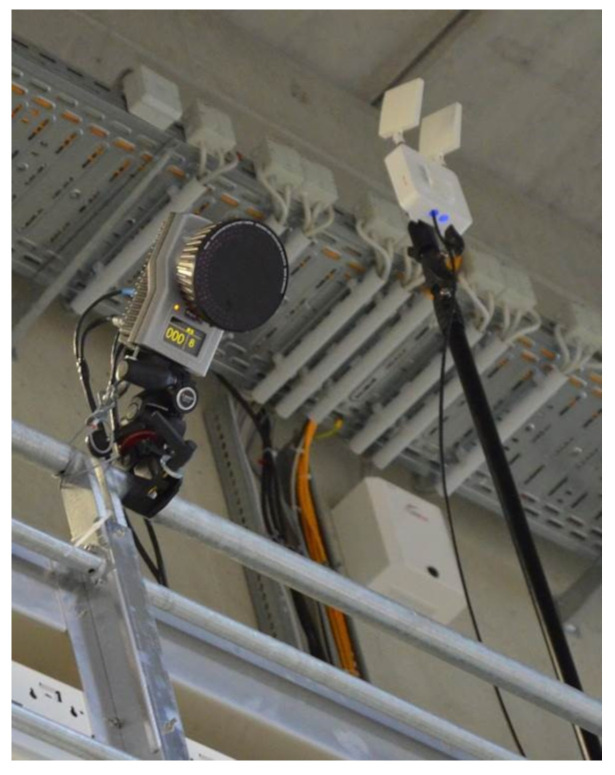
Balcony-mounted Qualysis camera (left) and Kinexon antenna (right).

**Figure 2:**
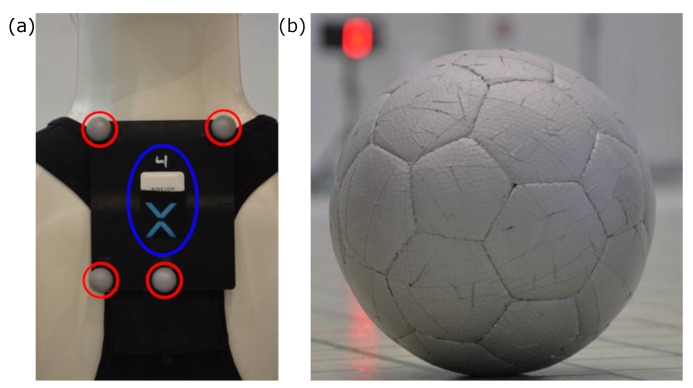
(**a**) Kinexon tag attachment (blue circle) and reference system marker arrangement (red circles). (**b**) Ball as reflective reference system marker with Kinexon sensor inside.

A commercially available LPS (KINEXON Precision Technologies, Munich, Germany) was investigated in this study. Firmware versions and application software versions corresponded to the latest releases on the testing date (APP version: 7.11.21, Stream processor version: 7.11.2). The installation and calibration of the system was guided by technicians of the manufacturer. Around the playing field 26 antennas and two base stations were evenly distributed at three different height levels above the ground (Figure 1). The calibration required the exact assessment of all antennas’ 3D positions with respect to the local measurement area, using a Tachymeter with millimetre accuracy. With the help of these reference positions, the LPS determines the location of the player and ball tags. This calibration procedure is necessary if the system is installed at a new place. The different height of the assembled antennas enabled 3D measurements of the ball tags. The player tag was positioned between each player’s shoulder blades utilizing a pouch sewn into the player’s jersey (Figure 2).

The sensors transmitted time information via radio-technology to the antennas, which then forwarded the signal via a wide local area network to the local static base stations. Afterwards, the time information of all antennas were aggregated by a central computer and combined into positional data. The momentary position of a player was determined with a frequency of 20 Hz. Certified handballs and footballs were equipped with sensor tags underneath the spherical surface. This arrangement is similar to what is approved in professional leagues (Figure 2).

### 2.3. Testing Site and Reference System

**Figure 3:**
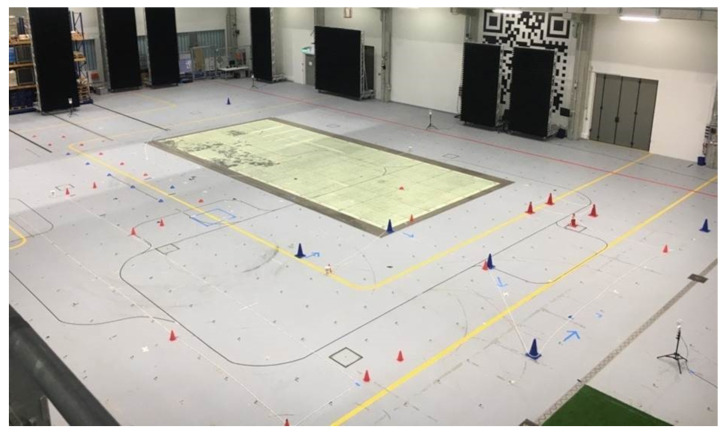
Test setup at the Fraunhofer L.I.N.K test hall in Nuremberg.

The test took place in the test and application center L.I.N.K. (Figure 3) at the Fraunhofer Institute for Integrated Circuits (Nuremberg, Germany). The setup covered an area of 26 × 16 × 6 m (base area: 416 m^2^) for measurements with both systems. The size of the field was limited by the dimension of the measurement hall. All cameras of the reference system were mounted on a gallery above the measurement area.

Criterion positions for dynamic accuracy determination were captured by a 30-camera motion capture system (28 Oqus 700+ cameras, 2 Miqus Video cameras, Qualisys, Sweden; Figure 1). Based on infra-red determination of reflective markers a precise calculation of the 3D-positions of the markers with a sample rate of 120 Hz was achieved.

To test the spatial accuracy of the reference system, a calibration object with known dimensions was moved within the measurement area [8]. Deviations of the known spatial distance between the markers and distance measured by the motion tracking system resulted in a mean deviation of 2.89 mm (SD = 1.66 mm, 95% CI [−3.22 mm, +3.27 mm]). The root mean square error (RMSE) was 1.7 mm.

For player tracking, several markers were placed on the upper thoracic spine between the scapulae (Figure 2). The software recognized the different marker patterns for each player and automatically calculated the center as current tracking position (Qualisys track manager 2019.3).

Both, handballs and footballs were completely covered with reflective foil. This enabled the reference system to track the ball as a single object, meaning the center of the tracked marker is corresponding to the center of the ball (Figure 2).

### 2.4. Testing Protocol and Sample Size

The test setup contained both handball and football specific exercises to cover a variety of game sport relevant situations.

**Figure 4:**
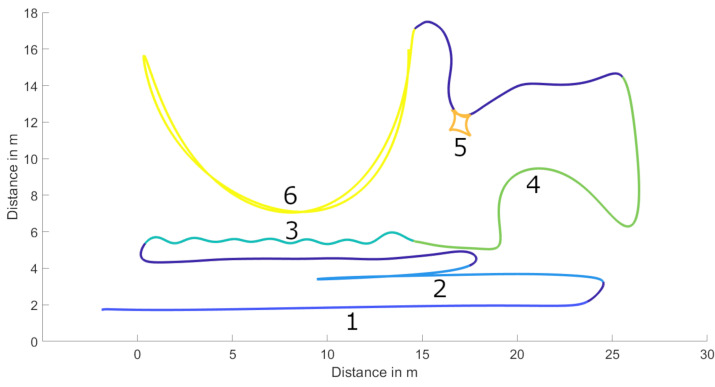
Sport-specific course (SSC) example with Qualisys data. Exercises in chronological order: 1 = linear sprint; 2 = 505 agility test; 3 = zig-zag jogging; 4 = squat jump and sharp changes of direction; 5 = multi-directional lunges; 6 = two curved sprints

All participants conducted four trials of a sport-specific course (SSC), containing typical exercises of team sports. The elements were selected to test different critical capabilities of player position tracking in high speed, acceleration and changes of direction (COD) periods [19]. The exercises and intensities used are common practice for testing the accuracy of position detection systems in sports [8,11,17,20,22]. The course consisted of a linear sprint (1), 405 agility test (2), zig-zag jogging (3), squat jump (SJ) followed by sharp COD (4), multi-directional lunges (5) and two curved sprints (6). An exemplary trajectory of the course is shown in Figure 4.

To test game specific patterns 4 vs. 4 and 3 vs. 3 small-sided football games (SSG) without goals were conducted within the test area. Each game lasted for 2 min, followed by 1 min of passive rest. The players followed the aim of keeping ball possession within the team and were instructed to keep the intensity at a high level. If the ball left the playing field, it was immediately returned by assistants around the field. This ensured a high net playing time in the SSGs.

Ball shots and throws were tested in 46 football 11-meter penalty kicks and 72 handball 7-m throws without a goalkeeper. A 2 × 3 m goal was placed at the respective distance. As this study aims only at validation, the same setup was used for shots and throws. The participants were instructed to distribute the shots and throws equally over the whole area of the goal. In total, 36 of the 7-m throws were executed as bounced shots.

Table 1 shows the sample size divided into player and ball tracking. For player tracking no trial had to be removed, which resulted in a total sample size of 32 SSC and 36 SSG trials. Ball positions were acquired for all 6 SSG, 46 football penalty shots and 71 handball seven-meter throws. The handball throws altered between direct and bounced shots. All SSG and shot trials were included in the analysis. One throw had to be excluded, due to synchronization problems. In the post processing of the data, tracking errors of the reference system such as out-of-bounds sample points were excluded from the analysis. Shots and throws had a very short duration, thus time and frame data are omitted in Table 1.

### 2.5. Data Processing

Position data of both systems were exported as raw data to local text files. Data of the Kinexon LPS-System was sampled at 20 Hz for players and 50 Hz for the ball. Reference system data was sampled with a frequency of 120 Hz. All further steps were executed in MATLAB (R2019b, The MathWorks Inc., Natick, MA, USA). The criterion data was downsampled to the Kinexon sample frequency, using a linear interpolation algorithm.

Raw positional data of all players were filtered with a fourth order Butterworth low pass filter. The filtering method was adopted from validation studies with similar exercises [20]. In previous studies an appropriate cut-off frequency of 1 Hz was determined by analysing occurring gait frequencies of football players with a method described by Winter [29]. Raw ball positions were filtered with a 4th order Butterworth low pass filter and a cut-off frequency of 10 Hz.

Many use cases in sports require the analysis of speed and acceleration. Most commercial systems provide these variables in their output. However, to assure better comparability in this study, the filtered positional data were used as basis for the calculation. Speed (rate of change in XY position) and acceleration (rate of change in speed) were derived by differencing two consecutive data points. This procedure was applied to player as well as ball data. Peak speed, peak acceleration and peak deceleration represents the maximum or minimum momentary value in the respective data.

The alignment of both signals was accomplished in two steps. Initially all trials were synchronized temporally. Therefore, the system data was time-shifted until the minimal total RMSE between the speed values was found. For spatial synchronisation, a Procrustes analysis (Euclidean similarity transformation) was conducted to find the best fitting rotational and translational parameters and align both systems.

3D ball accuracy was investigated by comparing the tracked impact positions of shots and throws crossing the goal line. The start and end of each shot and throw were manually tagged. The intersection point of the ball with the goal plane was calculated using the manually defined start position and the closest tracked position to the goal plane. The coordinates of the goal were measured manually and did not change in during whole test. Position errors are recorded in 2D (XY), height (Z) and 3D (XYZ).

As tracking devices are usually meant to show the gross movement of a player and to avoid overestimation of covered distances by body sway when standing still, the data was gait neutralized before the distance covered was calculated [8]. Waypoints were created every 60 cm, the positions in between were interpolated using a shape-preserving piecewise cubic spline algorithm [8]. Finally the distance between each frame was summarized, resulting in the total distance covered. In addition to the total value, distances are given in speed zones, using the following thresholds: Zone 1 (<6 km·h^−1^), Zone 2 (≥6 to <15 km·h^−1^), Zone 3 (≥15 to <20 km·h^−1^), Zone 4 (≥20 to <25 km·h^−1^), Zone 5 (≥25 km·h^−1^).

### 2.6. Statistical Analysis

Measurement errors of positional data are stated by means of root mean square error (RMSE) and mean absolute error (MAE):

(1) $$\mathrm{RMSE}=\sqrt{\frac{\sum_{i=1}^{n}{\left(\mathrm{measured}\;\mathrm{distance}-\mathrm{actual}\;\mathrm{distance}\right)}^{2}}{number\;of\;measurements}}$$

(2) $$\mathrm{MAE}=\sum_{i=1}^{n}\left|measured\;distance-actual\;distance\right|$$

Both error indicators are stated for three variables. Position (m): Position RMSE and MAE, Speed (m·s^−1^): Instantaneous speed RMSE and MAE and Acceleration (m·s^−2^): Instantaneous acceleration RMSE and MAE. For position measurements, the circular error propable (CEP) as the median and the CE95 as the the 95th percentile of error values are calculated.

Three indications of percentage differences occure in the results: Tables 3 and 5 include the percentage difference for measured peak speed, peak acceleration and peak deceleration values. Table 3 also shows the absolute percentage difference; Table 4 shows the absolute percentage difference for shot and throw position errors; Table 6 elaborates normal and absolute percentage differences between measured covered distance in the speed zones. Speed zones 4 and 5 are excluded (-) for SSGs, as only two athletes reached zone 4 and none reached zone 5. Percentage deviation is calculated for covered distances above 0 m in both systems. In both cases, the omitted covered distances are added to the total distance.

All differences are stated as the average percentage deviation of the former variable in the respective trials.

## 3. Results

The results are structured in three different sub-sections: Position, speed and acceleration (1), Peak values (2), and Shot and throw tracking (3). All table values are rounded after calculation which can lead to small inconsistencies in printed outcomes.

### 3.1. Position, Speed and Acceleration

Table 2 presents the 2D deviations of momentary position, speed and acceleration between the reference system and Kinexon.

### 3.2. Peak Speed, Peak Acceleration and Peak Deceleration

Table 3 shows the mean and standard deviation (SD) of peak speed, peak acceleration and peak deceleration for the different stages of the SSC. Differences between both systems are shown as relative and absolute percentage deviations.

### 3.3. Shot and Throw Tracking

Deviations of the tracked position the ball passing the goal line are show in Table 4, Table 5 shows the measured ball speed peaks in shots and throws.

### 3.4. Covered Distance

Table 6 presents the covered distance measured by the reference system and Kinexon.

## 4. Discussion

### 4.1. Discussion of Results

The RMSE of 9 cm for positional measurements for the whole course did not differ largely from the RMSE of 8 cm for SSG tracking. Both mean absolute errors turned out to be 1 cm smaller (Table 2). The small advantage in accuracy of SSG compared to SSC is to be expected as SSC contains more critical events for position detection. Table 7 shows the accuracy of player position tracking from studies using a similar reference system.

A similar result pattern was found for general speed and acceleration errors. Looking at the specific exercises of the course, tracking in high velocity phases (e.g., linear and curved sprints) was less accurate compared to other sections (Table 2). Small differences between RMSE and MAE (Table 2) hint towards a constant tracking error instead of several peak errors, as calculation of RMSE squares all errors before taking the mean and the root.

Table 6 presents the difference of measured covered distance, divided into five speed zones and the total value. In total, the percentage deviation was 0.6% for SSCs and 1.0% for SSGs. The positive differences indicate, that all covered distances were slightly overestimated by the Kinexon system. These results are comparable to other studies investigating LPS [11,20,23].

Table 3 demonstrates errors in peak speed, peak acceleration and peak deceleration and the percentage deviations between Kinexon’s and Qualisys’s measured values. The percentage deviation of measured peak speed in the six exercises of the course were in the range of 0.7–1.7%. The error ranges got bigger for acceleration (−1.3–10.2%) and deceleration (−0.3–4.3%). The exercises with the highest percentage peak speed deviation were zig-zag jogging and the squad jump followed by CODs. Additionally, a high peak acceleration discrepancy was found for zig-zag jogging. The linear sprints with the highest peak speed also showed an overestimation by the LPS. Such overestimations can be found in other studies’ results, assessing Kinexon’s peak speed measurements. Fleureau et al. mention a mean bias of 0.15 (m·s^−1^) and 0.17 (m·s^−1^) for side- and center-field sprints [24]. These results indicate difficulties with the system in the assessment of speed and acceleration for alternating trajectories and are in line with similar shortcomings of LPS systems stated for LPS systems [19].

The 2D tracking accuracy of the football showed a position RMSE of 15 cm for SSGs. Compared to player tracking accuracy, this turns out to be almost twice as high (player tracking RMSE 8 cm; Table 2). Even the increased sampling frequency of 50 Hz (ball) compared to 20 Hz (player) could not compensate for that. This is to be expected, because the ball shows more critical kinematics, e.g., acceleration and speed than players’ movements. Additionally, the systematic error mentioned, caused by the location of the LPS sensor in the ball, influences the results. For ball tracking, the error should be interpreted respecting the intended usage of the data. Although the positional error of ball tracking was higher then the error found for player tracking in this study, the height of this error was stated as acceptable for player tracking in other studies [17]. Therefore, ball tracking accuracy could be appropriate for purposes like ball possession analysis [28]. For officiating purposes such as hawkeye in tennis, where error rates of well under 1 cm are achieved [30] the ball tracking should not yet be used.

Table 4 shows a deviation of 17 cm (shot) and 21 cm (throw) between the tracked 3D ball impact locations in the goal. This is a difference of 18.9% between the tracking of handballs and footballs. When looking at the composition of the error, rather big differences occur in height measurements, whereas the 2D position error was 13 cm for both ball types. In total, ball position tracking was more error prone than player tracking. The measured peak speed of the ball in shots and throws was slightly overestimated by the LPS (Table 5). This discrepancy was in the same range for both balls.

### 4.2. Discussion of Methods

There are different designs for validation studies of a position tracking system [19]. As Luteberget and Gilgien [12] mention, a gold standard design is indispensable and preferential to just comparing results of position tracking systems. A 3D-motion capture system based on infrared cameras with passive markers may be seen as the presently most effective reference system [12].

In this study, different stations of a sport-specific course were chosen to mimic relevant movements in team sports, with a focus on critical situations for position tracking devices. However, not all common and imaginable movements could be integrated. This should be taken into account, especially for sports with focus on other movement patterns.

The data processing in this study was chosen to be appropriate and applicable to recordings of two individual systems. However, the adaptation of the data processing to one specific system might be beneficial for optimizing this system’s results. Differential filtering settings are commonly used by manufacturers to improve results. This filter adaptation might result in more precise tracking outcomes [9].

Player tracking was evaluated using a course with different sections and small sided games. The course was designed to cover exercises for various demands within the testing area. This area was limited to the coverage capacity of the reference system. To the best knowledge of the authors, currently available infrared camera-based motion capture systems cannot cover large areas like a whole handball or even football pitch. Nevertheless, the size of the pitch has a significant influence on player kinematics and tactical behaviour. Neither did the relatively short track for linear sprints allow for reaching top sprinting speed nor did the limited pitch dimensions of the SSG allow for reaching top speeds comparable to full pitch handball or football matches. Moreover, for the same reason only a small number of samples in high or very high speed sections could be compared. This can be seen in the low availability of high speed covered distance in SSCs and the lack of high speed occurrences in SSGs (Table 6). Although this limitation is caused by the state of the art in validation methodology, it has the unpleasant consequence that we are not able to validate systems in speed zones, where accuracy gets increasingly critical.

Ball tracking accuracy for handballs and footballs is demonstrated in 2D (Table 2) and 3D (Table 4). Shots and throws were conducted with a large distribution of height differences and therefore allowed the analysis in 3D. Here, the Z-coordinate (height of the ball) was taken into account. In SSGs, the players’ aim of keeping ball possession led to the passes predominantly with the ball on the ground. Ball position measurements in SSGs were compared in 2D. The height accuracy in game-like scenarios should be more specifically addressed in further studies.

The tracking of the ball depends, among other things, on the positioning of the sensor, which is right underneath the surface of the ball. For a football (around ø 22 cm) and a handball (around ø 18 cm), this results in a maximum dislocation of 11 cm/9 cm to the center of the ball in the XY plane. As the ball is spinning, there is a variable systematic error ranging from 0 cm to 11 cm. This error is part of the deviation between reference and tested system, but may not really be seen as a measurement error.

## 5. Conclusions

This study investigated the sport-specific validity of Kinexon’s LPS for both, player and ball tracking data. The comparison with an infrared camera-based motion tracking system as criterion reference system allows for precise and continuous evaluation of the tracking accuracy. The exercises in the course were chosen to reflect typical movements of athletes in team sports, especially handball and football. It has to be considered, that the coverage area of the reference system falls short of original handball and football pitches thus not allowing for long phases of high velocities and coverage of a real-sized pitch.

The position measurements of players in the SSC showed just minor differences within all exercises, depending on position, speed and acceleration. For the game-like test setting, the error was slightly lower. These results are in line or even better compared to previous studies on LPS or video-based systems and better than GPS. Ball tracking showed a higher error than player tracking. The 3D accuracy for shots and throws depends on measurements in the Z direction and shows differences between handballs and footballs. The speed of the ball was slightly overestimated by the LPS.

Based on the results of this study, the accuracy of Kinexon’s LPS represents the current state of the art regarding player and ball position detection in handball and football. The system can be confidently used to track player and ball positions in team sports.

## Figures and Tables

**Table 1:** Overview of trials and sample points, used for player and ball tracking in SSCs and SSGs.

	Player Tracking		Ball Tracking		
	**SSC**	**SSG**	**SSG**	**Shots**	**Throws**
Trials valid	32	38	6	46	71
Trials excluded	0	0	0	0	1
Sample points valid	55,783	83,086	24,374	-	-
Sample points excluded	2147	4788	7230	0	0
Net time (min)	46.5	69.2	8.1	-	-

**Table 2:** Position, speed and acceleration errors measured for SSC and SSG. For each category, root mean square error (RMSE) and mean absolute error (MAE) as well as their standard deviation (SD) is shown. CEP indicates the median position error, CE95 the 95th percentile of error values.

		Position (m)		Speed (m·s^−1^)		Acceleration (m·s^−2^)			
		RMSE ± SD	MAE ± SD	CEP	CE95	RMSE ± SD	MAE ± SD	RMSE ± SD	MAE ± SD
SSC	Total	0.09	0.08	0.07	0.15	0.07	0.05	0.20	0.12
		0.02	0.01			0.01	0.01	0.05	0.01
	Sprint	0.13	0.12	0.12	0.19	0.11	0.09	0.18	0.14
		0.03	0.02			0.04	0.03	0.07	0.05
	405-Agility	0.08	0.07	0.07	0.13	0.07	0.06	0.25	0.15
		0.03	0.02			0.03	0.02	0.17	0.05
	Zig-Zag	0.07	0.06	0.06	0.10	0.04	0.03	0.10	0.08
		0.02	0.02			0.01	0.01	0.02	0.02
	SJ + sharp turns	0.08	0.07	0.07	0.13	0.07	0.05	0.18	0.12
		0.01	0.01			0.02	0.01	0.05	0.03
	Lunges	0.08	0.07	0.07	0.12	0.06	0.05	0.28	0.19
		0.02	0.02			0.02	0.02	0.14	0.09
	Curved sprints	0.11	0.10	0.10	0.17	0.10	0.07	0.31	0.16
		0.03	0.03			0.03	0.02	0.14	0.05
SSG	Player	0.08	0.07	0.6	0.13	0.06	0.04	0.18	0.10
		0.01	0.01			0.02	0.01	0.04	0.02
	Ball	0.15	0.12	0.11	0.22	1.61	0.86	36.06	19.22
		0.03	0.02			0.75	0.09	14.57	2.21

**Table 3:** Table with peak speed, peak acceleration and peak deceleration of the reference and Kinexon system for different parts of the SSC. Percentage values indicate the differences between both systems.

	Peak Speed (m·s^−1^)				Peak Acceleration (m·s^−2^)				Peak Deceleration (m·s^−2^)			
	Ref Sys ± SD	Kinexon ± SD	% Diff	Absolute % Diff	Ref Sys ± SD	Kinexon ± SD	% Diff	Absolute % Diff	Ref Sys ± SD	Kinexon ± SD	% Diff	Absolute % Diff
Linear Sprint	7.43	7.51	1.0%	1.0%	3.82	3.76	−0.5%	5.2%	−4.64	−4.75	2.4%	2.4%
	0.39	0.40			0.62	0.48			0.49	0.51		
405 Agility	5.90	5.98	1.4%	1.4%	7.01	6.92	−1.3%	2.7%	−7.45	−7.42	−0.3%	2.1%
	0.32	0.32			0.57	0.52			0.44	0.41		
Zig-Zag	1.66	1.69	1.7%	2.4%	0.91	1.00	10.2%	12.9%	−1.02	−1.03	−0.1%	7.3%
	0.16	0.16			0.33	0.35			0.33	0.35		
SJ and COD	4.43	4.51	1.7%	1.7%	4.52	4.63	2.4%	3.1%	−4.58	−4.70	2.7%	3.4%
	0.35	0.36			0.49	0.51			0.47	0.50		
Lunges	1.21	1.22	1.5%	3.2%	1.99	2.04	2.9%	5.7%	−2.01	−2.09	4.3%	6.1%
	0.28	0.27			0.62	0.61			0.55	0.54		
Curved sprints	5.82	5.86	0.7%	0.8%	6.08	6.10	0.5%	3.0%	−6.64	−6.71	1.1%	2.5%
	0.22	0.21			0.50	0.49			0.41	0.38		

**Table 4:** Football shot and handball throw: Deviation of impact position at the goal. The percentage difference states the deviation between shot and throw errors.

	ShotPos Error ± SD (m)	ThrowPos Error ± SD (m)	% Diff
2D	0.13	0.13	2.4%
	0.08	0.15	
Height	0.09	0.15	38.7%
	0.07	0.13	
3D	0.17	0.21	18.9%
	0.08	0.18	

**Table 5:** 2D peak speed of shots and throws.

	Ref Sys ± SD (m·s^−1^)	Kinexon ± SD (m·s^−1^)	% Diff
Shot	24.65	25.05	1.8%
	3.49	3.50	
Throw	17.78	18.20	2.6%
	1.64	2.27	

**Table 6:** Covered distance in SSCs and SSGs, shown in five speed zones and the total trial. In SSGs, zone 4 was only reached in two occasions and is therefore excluded. No player reached speed zone 5 in all SSGs.

		Ref Sys ± SD (m)	Kinexon ± SD (m)	% Diff	Absolute % Diff
SSC	Total	173.9	174.9	0.6%	0.6%
		6.7	6.8		
	Zone 1	48.8	48.9	0.2%	1.3%
		7.8	7.9		
	Zone 2	50.1	50.3	0.3%	1.3%
		8.5	8.8		
	Zone 3	46.7	45.0	−3.7%	4.1%
		7.2	7.5		
	Zone 4	21.2	22.7	8.5%	9.1%
		8.9	9.1		
	Zone 5	7.2	8.0	14.5%	15.5%
		3.9	3.8		
SSG	Total	165.5	167.1	1.0%	1.0%
		27.4	27.6		
	Zone 1	61.5	61.2	−0.5%	1.4%
		10.4	10.6		
	Zone 2	98.3	99.6	1.4%	1.7%
		29.2	29.4		
	Zone 3	5.4	6.0	15.3%	16.5%
		6.6	7.0		
	Zone 4	-	-	-	-
	Zone 5	-	-	-	-

**Table 7:** Results from studies using similar validation procedures.

Article	Tested System	Reference System	Exercises	Result
Ogris et al. [2012] [22]	LPS	Vicon	Courses, SSG	MAE: 23.4 cm
Linke et al. [2018] [8]	GPS, LPS, VID	Vicon	Courses, SSG, Shuttle runs	RMSE GPS: 96 cm RMSE LPS: 23 cm RMSE VID: 56 cm
Luteberget et al. [2018] [11]	LPS	Qualisys	Courses	MAE: 21 cm
Linke et al. [2020] [20]	2 × VID	Vicon	Courses, SSG	RMSE VID1: 9 cm RMSE VID2: 8 cm
Hodder et al. [2020] [17]	LPS	Vicon	Courses	RMSE: 20 cm

## Data Availability

The data presented in this study are available in the article.
